# Density-based clustering of crystal (mis)orientations and the *orix* Python library

**DOI:** 10.1107/S1600576720011103

**Published:** 2020-09-23

**Authors:** Duncan N. Johnstone, Ben H. Martineau, Phillip Crout, Paul A. Midgley, Alexander S. Eggeman

**Affiliations:** a Department of Materials Science and Metallurgy, University of Cambridge, 27 Charles Babbage Road, Cambridge CB3 0FS, United Kingdom; b Department of Materials, The University of Manchester, Oxford Road, Manchester M13 9PL, United Kingdom

**Keywords:** data clustering, crystal orientations, fundamental zones, computer programs, Python

## Abstract

Crystal orientation mapping experiments typically measure orientations that are similar within grains and misorientations that are similar along grain boundaries. Such (mis)orientation data cluster in (mis)orientation space, and clusters are more pronounced if preferred orientations or special orientation relationships are present. Here, cluster analysis of (mis)orientation data is described and demonstrated using distance metrics incorporating crystal symmetry and the density-based clustering algorithm DBSCAN. Frequently measured (mis)orientations are identified as corresponding to similarly (mis)oriented grains or grain boundaries, which are visualized both spatially and in three-dimensional (mis)orientation spaces. An example is presented identifying deformation twinning modes in titanium, highlighting a key application of the clustering approach in identifying crystallographic orientation relationships and similarly oriented grains resulting from specific transformation pathways. A new open-source Python library, *orix*, that enabled this work is also reported.

## Abstract 2

Data clustering incorporating symmetry is applied to crystal orientations and misorientations and the *orix* Python library for crystal orientation analysis is introduced.

## 1. Introduction   

**Figure 1:**
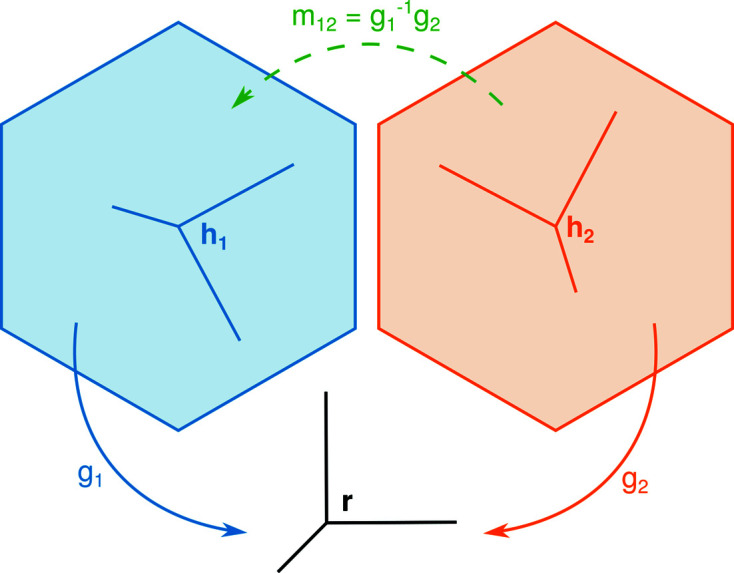
Schematic representation of orientations, 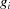, and misorientations, *m*, as transformations between reference frames.

The distribution of crystal orientations in a polycrystalline material (*i.e.* crystallographic texture) and characteristic misorientations between neighbouring crystals (*i.e.* orientation relationships) are affected by material processing and influence material properties (Kocks *et al.*, 1998 ▸; Sutton & Baluffi, 2007 ▸). Measuring the local crystal orientation throughout a material is therefore common in modern materials characterization. Such mapping is usually achieved using scanning diffraction techniques such as electron backscatter diffraction (EBSD) (Schwartz, 2009 ▸), scanning electron diffraction (Zaefferer, 2000 ▸; Rauch *et al.*, 2008 ▸) and X-ray microLaue diffraction (Ice & Pang, 2009 ▸). These techniques use a small (nm–µm) probe to address numerous locations across the specimen while recording diffraction data at each position. Such data can be used to determine the local crystal ‘orientation’, conventionally defined^1^ (Rowenhorst *et al.*, 2015 ▸) as the passive rotation, 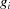, between the crystal coordinate system, 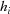, and a reference specimen coordinate system, *r* (Morawiec, 2004 ▸), *i.e.*

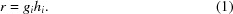
Determining the crystal orientation at each two-dimensional pixel or three-dimensional voxel produces a crystal orientation map. The misorientation, *m*, between crystals at two locations is then the passive rotation between crystal coordinates,
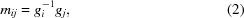
where 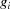 and 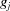 are the orientations of each crystal, as illustrated in Fig. 1 ▸. Since crystal orientations and misorientations are both described as passive rotations in three dimensions, they can be represented and analysed similarly provided that crystal symmetry is treated appropriately.

Crystal (mis)orientations may be represented as vectors in three-dimensional neo-Eulerian vector spaces based on parametrization of the corresponding axis and angle of rotation (Frank, 1988 ▸, 1992 ▸). Visualizing (mis)orientation data within the symmetry-reduced fundamental zone (or asymmetric domain) of such spaces has recently become more accessible owing to the availability of open-source software packages (Bachmann *et al.*, 2010 ▸; Groeber & Jackson, 2014 ▸). Clusters of (mis)orientations are typically observed within the fundamental zone because (mis)orientation measurements within an individual grain or along a grain boundary are similar. Furthermore, measurements from multiple crystals add to the same cluster if there are preferred crystal orientations or special orientation relationships. Identifying clusters in (mis)orientation data therefore provides a route to identify grains and grain boundaries as well as preferred crystal orientations and orientation relationships. This approach has recently been used to identify grains and crystallographic orientation relationships via the manual identification of (mis)orientation clusters (Callahan *et al.*, 2017 ▸; Krakow *et al.*, 2017*b* ▸,*c* ▸; Sunde *et al.*, 2019 ▸). However, clusters that cross fundamental zone boundaries appear split as a result of the crystal symmetry relating the boundaries, which makes the visualization less clear (Krakow *et al.*, 2017*b* ▸). This motivates a computational approach to (mis)orientation cluster analysis, both to remove manual steps and to improve visualization.

Clustering of crystal orientations must account for crystal symmetry, which implies that a (mis)orientation is only known up to the action of elements of the proper point group (Krakow *et al.*, 2017*b* ▸). Recently a number of authors have considered the statistics of such ambiguous rotations (Arnold *et al.*, 2018 ▸; Chen *et al.*, 2015*a* ▸; Niezgoda *et al.*, 2016 ▸), and hierarchical clustering of (mis)orientations in the presence of crystal symmetry has been demonstrated (Krakow *et al.*, 2017*a* ▸). Furthermore, a model-based clustering algorithm accommodating symmetry, based on a mixture of von Mises–Fisher and Watson distributions and with parameters estimated using expectation maximization, has also been reported for orientations (Chen *et al.*, 2015*a* ▸,*b* ▸). In this work, we report on density-based clustering of (mis)orientations in the presence of crystal symmetry and establish an open-source Python library, named *orix*, for handling crystal (mis)orientation data.

## 2. The *orix* Python library   

Here, we describe *orix-0.2.3* (released May 2020), which defines various classes and methods that enable (i) calculations to be performed with three-dimensional rotations, (ii) the application of crystal symmetry to rotations for all proper point groups and (iii) the visualization of (mis)orientations in three-dimensional neo-Eulerian vector spaces (Krakow *et al.*, 2017*b* ▸). All rotation calculations are performed in the quaternion representation and conversions between common representations, including Euler angles and axis–angle pairs, are supported (Rowenhorst *et al.*, 2015 ▸).

The passive rotation convention defined by equation (1) and the axis alignment conventions set out by Krakow *et al.* (2017*b* ▸) are adopted for (mis)orientations in *orix*. The (mis)orientation data must therefore be converted to these conventions if the data are represented in the active rotation convention or with alternative axis alignments. Often the raw orientation mapping data will be expressed as an array of Euler angles output by automated indexing software. In this case, an orix.Rotation object can be initialized in the correct orix convention, starting from most common conventions (Rowenhorst *et al.*, 2015 ▸), using the from_ euler() method.

*Orix* is released open source (Crout *et al.*, 2020 ▸) under the GPL-3 licence and depends only on core packages in the scientific Python stack, namely *NumPy* (van der Walt *et al.*, 2011 ▸), *SciPy* (Virtanen *et al.*, 2019 ▸) and *Matplotlib* (Hunter, 2007 ▸). The code is packaged on both the Python Package Index (PyPI; https://pypi.org/) and the conda-forge repository (https://conda-forge.org/) for use across Linux, Windows and OS X platforms. A comprehensive set of tests is packaged with the code, providing a strong platform for code maintenance and for further development of the package. Usage examples, including the methods described in this paper, are provided online (Johnstone & Crout, 2020 ▸) as a collection of *Jupyter* notebooks (Kluyver *et al.*, 2016 ▸).

The development of *orix* was heavily inspired by the much more extensive MATLAB toolbox *MTEX* (Bachmann *et al.*, 2010 ▸). We decided to establish a Python library in order to interface more easily with the wider scientific Python stack, for example enabling us to directly use clustering algorithms implemented in *scikit-learn* (Pedregosa *et al.*, 2011 ▸) in this work.

## 3. (Mis)orientation clustering method   

A cluster analysis is an attempt to partition a set of ‘objects’ 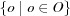, such as (mis)orientations, into a meaningful set, *K*, of subsets 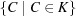 (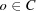), in which the ‘distance’ between objects within each subset *C* is less than the distance between objects in different subsets (Everitt *et al.*, 2011 ▸). To apply this broad definition, a metric for the distance, 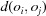, between two objects in the set must be defined such that the partition has ‘meaning’ and the conditions 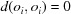 and 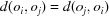 are satisfied (Everitt *et al.*, 2011 ▸). Furthermore, an appropriate clustering algorithm must be selected. Here, distance metrics for (mis)orientations including crystal symmetry and the suitability of density-based clustering algorithms for orientation mapping applications are explained.

### 3.1. Distance metrics for crystal (mis)orientations   

We define the distance, 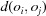, between two (mis)orientations as the minimum rotation angle relating them.^2^ This angle is symmetric, *i.e.* it is the same regardless of which orientation is the starting point, and zero for identical (mis)orientations, making it a suitable distance metric for clustering. The minimum rotation angle also has the significant advantage of being a physically intuitive distance metric, which makes subsequent clustering parameters similarly intuitive.

For crystal (mis)orientations, it is physical to consider symmetry equivalence. Crystal symmetry implies that the orientation of a crystal with proper point group symmetry, *S*, is equivalent following a transformation 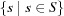. This crystal symmetry should be considered in order to determine the minimum rotational angle amongst symmetry-equivalent rotations and requires different treatment for orientations and misorientations. An orientation *g* is equivalent to the set of orientations defined by the equivalence group,
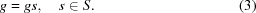
The rotation between orientations is a misorientation as defined by equation (2), and combining this definition with equation (3) yields an expression for symmetrically equivalent misorientations,
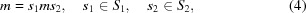
where 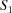 and 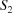 are the symmetry groups of the crystal in each orientation.

The distance between two orientations, 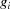 and 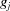, associated with crystals with the symmetry groups 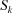 and 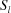, respectively, is thus given by
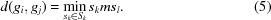
The distance between two misorienations is defined similarly as the rotation between two misorientations, 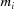 to 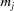, which, accounting for the crystal symmetry of the two pairs of crystals associated with each misorientation using equation (4), gives
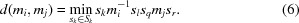
Here 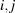 are indices indicating (mis)orientations associated with an orientation map and 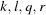 are indices indicating the symmetry group corresponding to the crystal phase associated with each (mis)orientation.

### 3.2. Density-based clustering of (mis)orientations   

A distance matrix, 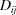, containing the distances between all (mis)orientations, may be defined using equations (5) and (6) and used to initialize a clustering algorithm. In clustering (mis)orientation data, we aim to identify an unknown number of small dense clusters associated with grains, grain boundaries and special orientation relationships while excluding spurious data points resulting from incorrect automated indexing. Density-based clustering methods are well suited to this application because they are based on identifying clusters as regions of higher density than the remainder of the data set while identifying points in sparse regions as noise or boundary points. This contrasts with centroid- and model-based methods that typically require a good estimate of the number of clusters and hierarchical clustering, which does not provide a unique partition and is not very robust to outliers (Everitt *et al.*, 2011 ▸). We note that model-based and hierarchical clustering methods have nevertheless been demonstrated to provide useful (mis)orientation clustering (Chen *et al.*, 2015*a* ▸; Krakow *et al.*, 2017*a* ▸).

We perform density-based clustering using the DBSCAN algorithm (Ester *et al.*, 1996 ▸) implemented in *scikit-learn* (Pedregosa *et al.*, 2011 ▸). This algorithm identifies clusters as regions containing a high density of data points separated by regions containing a low density of data points. Data points in high-density regions are identified as core samples, defined as data points within a distance ∊ of at least a minimum number *n* of other data points. A cluster is then determined by taking a core sample, expanding the cluster set to include all neighbouring data points within the distance ∊, identifying which of these data points are also core samples and recursively expanding the set around newly included core samples in the cluster. The cluster is eventually bounded by a set of non-core samples that are within the maximum distance ∊ of a core sample in the cluster but are not themselves core samples. Any data point that is not a core sample and is at a distance of at least ∊ from any core sample is considered an outlier, *i.e.* not part of any cluster. In contrast to other algorithms, for example the assumption of convex clusters in k-means clustering, the DBSCAN algorithm allows clusters to have any shape.

It is crucial that ∊ is chosen appropriately for the data set and distance metric. If ∊ is too small, most data points will not be included in any cluster. If ∊ is too large, close clusters will not be separated properly. A significant advantage of the distance metrics defined in Section 3.1 is that ∊ has an intuitive physical interpretation as the upper limit on the absolute rotation angle (in radians) between any data point and a core sample in a cluster. The parameter *n* primarily controls noise tolerance and should be increased for noisy or large data sets. Physically, this parameter sets a minimum number of spatial coordinates in a valid grain or grain boundary. In general, a range of parameters can be trialled to determine optimal values. In this work, we obtained reasonable results using ∊ = 0.05, *n* = 40 for orientations and ∊ = 0.05, *n* = 10 for misorientations.

## 4. (Mis)orientation clustering results   

An orientation map obtained via EBSD mapping of a commercially pure hexagonal close packed (h.c.p.) titanium (6/*mmm*, space group 194) sample, following high-strain-rate deformation, was used to illustrate the density-based (mis)orientation clustering method. This data set was downloaded from an online repository (Krakow & Hielscher, 2017 ▸) for this demonstration and was previously described in detail by Krakow *et al.* (2017*b* ▸). The orientation map contains data from two parent grains, each containing deformation twins.

### 4.1. Clustering orientations to find grains   

**Figure 2:**
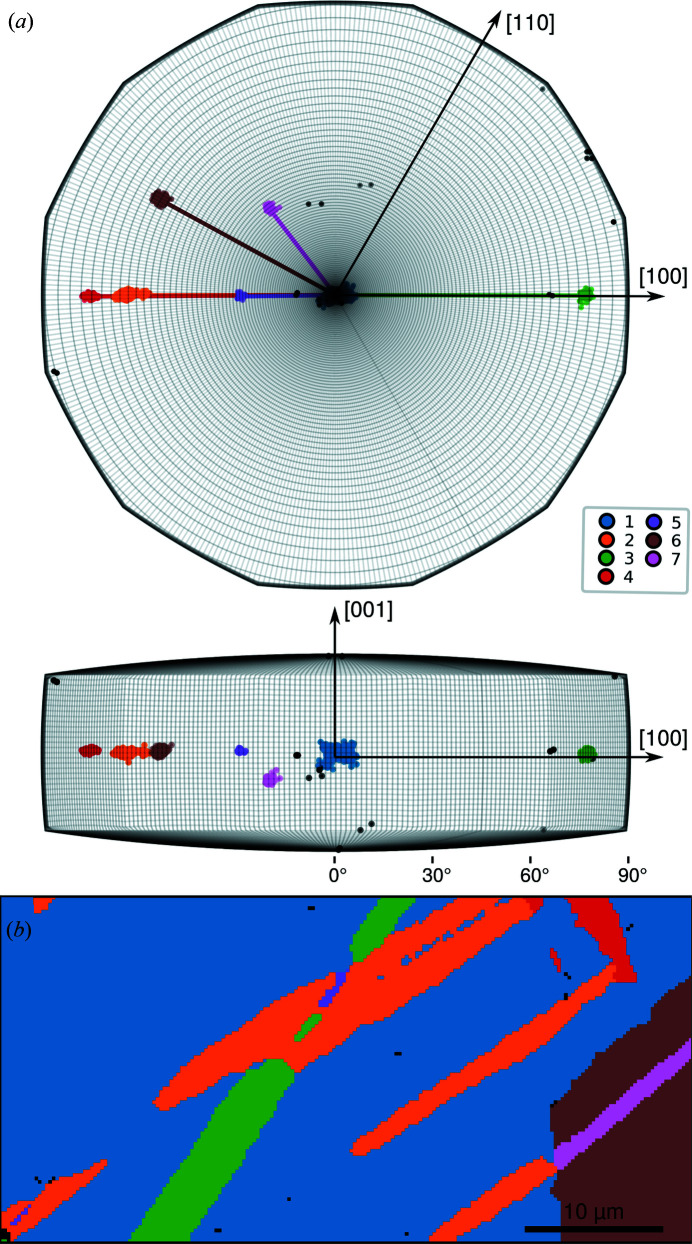
(*a*) Crystal orientations plotted within the fundamental zone for symmetry group 622 in axis–angle space and coloured to indicate cluster membership as determined using the DBSCAN algorithm. Axes are labelled in the crystallographic basis at no rotation. (*b*) Map of the twinned Ti microstructure coloured by cluster membership of the orientation associated with each pixel.

The orientation clusters determined by density-based clustering of the data are shown in Fig. 2 ▸(*a*). The clusters are plotted within the asymmetric domain of axis–angle space (Krakow *et al.*, 2017*b* ▸) for the proper point group, 622, of h.c.p. titanium and the mean orientation of the largest parent grain (cluster 1) is taken as the reference orientation. Clusters 2–5 are all rotated about [100] with respect to the reference parent grain (cluster 1), suggesting that they may correspond to twins, whereas clusters 6 and 7 are rotated about other axes.

Plotting the spatial location associated with data points in each orientation cluster, as shown in Fig. 2 ▸(*b*), provides a clear visualization of the grain structure and illustrates that the clustering result is physically meaningful. Clusters 2–5 correspond to lenticular grains, typical of deformation twins, within the larger parent grain (cluster 1). We note that similar twin variants are grouped together by the clustering analysis in cluster 2. Cluster 6 corresponds to the second parent grain and cluster 7 to a lenticular deformation twin within that grain. Some data points are not assigned to any cluster and correspond to automatically identified misindexed pixels. We note that despite the asymmetrical shape of some clusters (*e.g*. clusters 1 and 2) resulting from deformation within the grain this has not caused issues with this clustering.

### 4.2. Clustering misorientations at grain boundaries   

**Figure 3:**
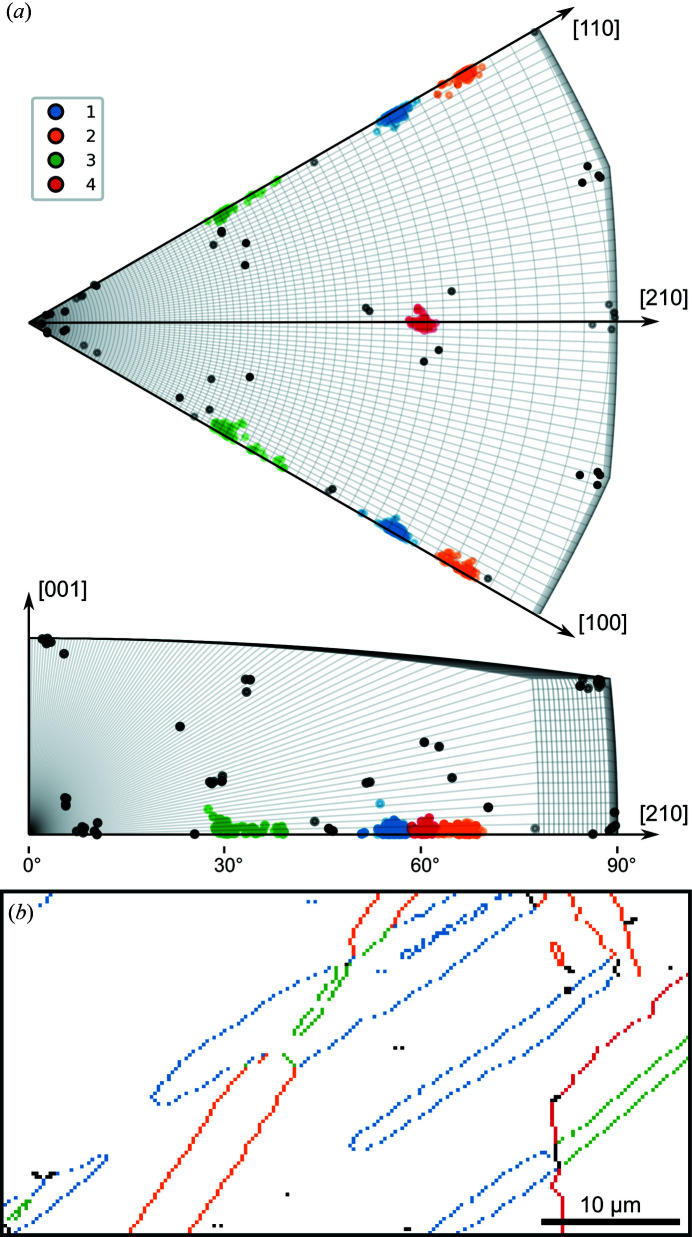
(*a*) Crystal misorientations plotted in the fundamental zone for the symmetry group pair (622, 622) in axis–angle space and coloured to indicate cluster membership as determined using the DBSCAN algorithm. Axes are labelled in the crystallographic basis at no rotation. (*b*) Map of grain boundaries coloured by cluster membership of the misorientation at each boundary element.

The misorientation between horizontally adjacent pixels was computed from the orientation mapping data. Misorientations with rotation angles less than 7°, corresponding to the grain orientation spread within the largest grain in this highly deformed material, were discarded in order to identify grain boundaries. The misorientation clusters determined by density-based clustering of these data are shown in Fig. 3 ▸(*a*). These misorientations are plotted within the asymmetric domain of axis–angle space for misorientations between two h.c.p. titanium crystals, each with proper point group symmetry 622, without application of grain exchange symmetry (Krakow *et al.*, 2017*b* ▸). Four clusters are identified, three of which (clusters 1–3) are situated across the boundary of the asymmetric domain and are identified as belonging to the same cluster owing to the inclusion of crystal symmetry in the distance metric.

The mean misorientation associated with each cluster highlighted in Fig. 3 ▸(*a*) was calculated as the quaternion mean (Morawiec, 1998 ▸) of misorientations in the cluster. The minimum rotational angle between these cluster centres and theoretical misorientations associated with near coincident site lattice (n-CSL) orientation relationships (Bonnet *et al.*, 1981 ▸), which result from deformation twinning (Lainé & Knowles, 2015 ▸), were computed to determine the closest n-CSL to each cluster centre. Clusters 1–3 were found to be within *ca* 1.2° of n-CSL relationships associated with deformation twinning, whereas cluster 4 was 4° from the nearest n-CSL relationship, as reported in Table 1 ▸. This suggests that clusters 1–3 correspond to deformation twin boundaries, whereas cluster 4 does not. Inspecting the spatial distribution of misorientation clusters, as in Fig. 3 ▸, confirms that clusters 1–3 correspond to deformation twin boundaries, whereas cluster 4 corresponds to the boundary between parent grains. All remaining points correspond to misindexed pixels. Some data points are not assigned to any cluster and correspond to automatically identified boundaries of misindexed pixels.

## 5. Discussion   

Density-based clustering using a distance metric that accounts for crystal symmetry has been demonstrated here to successfully characterize deformation twinning in experimental orientation mapping data. This includes treatment of spurious misindexed pixels and elongated asymmetrical clusters due to distortions within grains. The DBSCAN algorithm used here requires only two parameters to be set and therefore minimal prior knowledge. The clustering results enhance the practical utility of three-dimensional misorientation spaces as a tool for visualizing orientation mapping data by automatically identifying clusters. In particular, clusters that cross the boundaries of the fundamental zone are identified and can be indicated when plotting the data, making visualizations easier to interpret. Plotting the spatial distribution of (mis)orientation clusters further provides an easy way to relate observations in real space and (mis)orientation space.

The clustering analysis is not without limitations. Density-based clustering algorithms are known to struggle with data sets in which the overall density is high as a density drop is needed to identify cluster boundaries. This could occur when an orientation map contains data from a large number of grains, and in such cases an alternative solution may be more suitable, for example recently reported model-based clustering (Chen *et al.*, 2015*a* ▸,*b* ▸) or hierarchical clustering (Krakow *et al.*, 2017*a* ▸) approaches. As discussed in Section 3.2, these methods typically have the disadvantage of requiring an estimate for the number of clusters. A further limitation is that clusters are labelled but no parameters associated with the (mis)orientation distribution are estimated. Using the cluster centres as a starting point for fitting local (mis)orientation distribution functions may therefore be an important extension.

Physical insight is obtained by relating observed (mis)orientation clusters to special (mis)orientations, typically predicted via a crystal growth or deformation model. In the example presented above, this approach enabled identification of similar, though spatially separated, deformation twin variants and the corresponding active deformation twinning modes, based on predicted nCSL orientation relationships. This identification of (mis)orientation clusters that are consistent with hypothetical models could be extended by considering the probability of sampling the cluster from a random (mis)orientation distribution to assess statistical significance of the observed cluster. Furthermore, clustering analysis in (mis)orientation space does not use any spatial information and therefore groups spatially separated grains and grain boundaries with similar (mis)orientations. While this is sometimes the desired output, in other cases incorporating spatial information using conventional methods such as the ‘flood fill’ approach for grain identification may be preferable. Overall, we envisage the (mis)orientation clustering approach being most useful for validating crystal growth and deformation models as illustrated here.

## 6. Conclusions   

This work demonstrates that density-based clustering of crystal orientations and misorientations, using a distance metric accounting for crystal symmetry and the DBSCAN algorithm, can provide important physical insights using very little prior knowledge. In particular, we used this approach to identify characteristic misorientations associated with deformation twinning as an illustrative example of how the approach may be used to identify special orientation relationships and similar crystallographic transformation variants as key applications of the approach. A Python library, named *orix*, was established to provide various classes and methods required for the manipulation of (mis)orientation data, and it is hoped that this library will serve as a platform for further developments.

## Supplementary Material

orix source code: https://doi.org/10.5281/zenodo.3835880

orix demo notebooks: https://doi.org/10.5281/zenodo.3837261

## Figures and Tables

**Table 1:** Comparison of misorientation cluster mean values with near coincident site lattice misorientations (Bonnet *et al.*, 1981 ▸) calculated for titanium with an assumed *c*/*a* = 1.588

Cluster	Nearest n-CSL	Theoretical misorientation	Distance (°)
1	n-CSL7a	[100] 64.40°	0.44
2	n-CSL13a	[100] 76.89°	0.70
3	n-CSL11a	[100] 34.96°	1.19
4	n-CSL13b	[210] 57.22°	4.44

## Footnotes

1 It is also common for the active rotation convention to be adopted, *i.e.* for the crystal orientation to be defined as transforming the specimen reference frame into the crystal reference frame. For comments on dealing with orientation data represented using different conventions, see Section 2.

2 We note that an alternative approach could be to map the (mis)orientation data into a space with a uniform metric so that the Euclidean distance could be used. Such an approach could be computationally efficient but the corresponding parameters are less physically intuitive.
